# Radiation treatment with volumetric modulated arc therapy of hepatocellular carcinoma patients. Early clinical outcome and toxicity profile from a retrospective analysis of 138 patients

**DOI:** 10.1186/1748-717X-7-207

**Published:** 2012-12-08

**Authors:** Po-Ming Wang, Wei-Chung Hsu, Na-Na Chung, Feng-Ling Chang, Antonella Fogliata, Luca Cozzi

**Affiliations:** 1 Department of Radiation Oncology, Cheng-Ching General Hospital, Taichung, Taiwan; 2 Department of Healthcare Administration, Asia University, Taichung, Taiwan; 3 Oncology Institute of Southern Switzerland, Bellinzona, Switzerland

**Keywords:** Hepatocelluar Carcinoma, RapidArc, VMAT, Radiotherapy

## Abstract

**Background:**

To report early outcome and toxicity for inoperable patients with hepatocellular carcinoma (HCC) treated with volumetric modulated arc therapy (VMAT).

**Methods:**

One hundred and thirty eight patients were retrospectively analysed. Dose prescription ranged from 45 to 66Gy with conventional fractionation regime. Based on AJCC staging, 88.4% presented stage III or IV. Two-thirds (69.6%) were Child-Pugh stage A, the remaining were stage B. According to Barcelona Clinic Liver Cancer staging, 72.5% of patients were classified as stage C.

**Results:**

Median age was 66 years, median tumor volume was 516cm^3^ (28 to 3620cm^3^). The most patients (83%) were treated with 60Gy. Median follow-up time was 9 months. One-year overall survival rate was 45% (100% for AJCC stage I, 83% for stage II, 45% for stage III and 28% for stage IV), median survival was 10.3 months (95% C.I. 7.2-13.3). Local control was achieved in 94% (of 109 assessable patients), stable disease in 29%, partial response in 53%, complete response in 11%, and progression in 6%. Radiation-induced liver disease was observed in 34 patients (25%). Gastrointestinal grade 3 toxicity was modest with a total of 17 (12.3%) cases for all endpoints.

**Conclusions:**

Clinical results could suggest to introduce VMAT as an appropriate technique for the patients with HCC.

## Background

Hepatocellular carcinoma (HCC) is the third cause of cancer death and one of the most challenging oncological problems [1]. Surgery, although providing survival rates up to 70% at 5 years [2], is viable in a small fraction of patients (less than 1/3) because of advanced stage at diagnosis. Patients also can be treated with transcatheter arterial chemoembolization (TACE), radiofrequency ablation (RFA), percutaneous ethanol injection (PEI), chemotherapy and targeted agents [3-6] with complex decision trees and limited impact on outcome. Radiotherapy was offered to HCC patients but it was limited by severe radiation induced liver disease (RILD) when excessive fractions of the liver were involved in the radiation field [7] and the important relationship between the volume of irradiated normal liver and the toxicity profile [8-10]. After the introduction of intensity modulated radiotherapy (IMRT), a new hope emerged for radiotherapy in HCC patients [11,12]. Recently, IMRT evolved into the so-called volumetric modulated arc therapy (VMAT). VMAT was pioneered in its RapidArc mode by Otto [13] and technical details can be found elsewhere [14]. In liver, clinical application of RapidArc was primarily limited to metastatic indications [15]. A planning study from Kuo et al. demonstrated that RapidArc also play a role in HCC [16]. Stereotactic body radiation therapy (SBRT) approaches have been hypothesized for smaller target and in cases requiring modest irradiation of entire liver (less than 1/4 of the volume) [17].

Here, we report about the clinical application of RapidArc to HCC in a cohort of patients, demonstrating the feasibility and the early clinical outcome on a large patient population. Conventional fractionation and total dose were adopted because of the stage and the large median tumor size.

## Methods

### Patients

**Figure 1:**
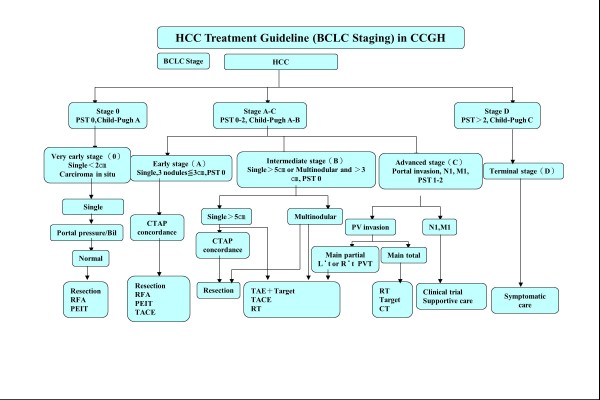
**Schematic representation of the institutional guidelines for the treatment management of HCC patients** .

Between February 2009 and December 2010, 138 consecutive HCC patients presented Barcelona Clinic Liver Cancer (BCLC) stage A to C and were eligible for curative or palliative radiotherapy (in eventual association to other therapeutic modalities) at the home institute. Figure 1 represents the institutional guidelines for HCC treatment. In brief, BCLC stages A to C, Child-Pugh stages A-B with single lesions larger than 5cm or multi-nodular lesions larger than 3cm were eligible for radiotherapy. All patients were inoperable or not eligible for TACE treatments and received radiotherapy as primary treatment. Portal vein thrombosis was present in about 50% of the cases. Relative contraindication to inclusion were: total bilirubin levels greater than 3 to 5 mg/dL; white blood count (WBC) less than 2500–1500 U/?L; Glutamic pyruvic transaminase (GPT) in the range 100–300 U/L. Absolute exclusion criteria included total bilirubin >5 mg/dL, WBC<1500 U/?L and GPT>300 U/L.

### Radiation treatment

Dose prescription was of 45, 60 or 66Gy in 1.8 or 2.0Gy/fraction depending upon stage, location of target and its size and general conditions of patient. Plans were designed for single course treatments of for sequential boosting on reduced volumes technique. In case of boost, three schemed were adopted: i) 60Gy total dose in two courses: 40Gy+20Gy; ii) 45Gy in two courses: 36Gy+9Gy; iii) 45Gy in three courses: 27Gy+9Gy+9Gy. Patients receiving a single course were treated at 66Gy. For those patients with tumor near gastrointestine tract, 45Gy in two or three courses were designed by the shrinkage of tumor and prevention for gastrointestine. The dosage and boost scheme were predetermined by the decisions of the physician. If the dose constraints couldn’t be satisfied, boost schemes were applied to decrease normal tissue complications. As an example, for existing duonenal ulcer, gastric ulcer, reflux esophagitis patients, 36+9Gy boost was applied to minimize radiation-induced ulcer. When OARs included stomach, duodenum and colon, the 40+20Gy scheme was applied. The gross tumor volume (GTV) was defined as the primary tumor plus abnormal portal areas revealed on CT images (IV contrast was used for all patients except for the patients with renal dysfunction. For those renal insufficient patients, MRI was added and fused to planning CT for more precise target delineation). The clinical target volume (CTV) was defined as the GTV plus a 1cm margin. The planning target volume (PTV) was defined as the CTV plus 0.5cm axial and 1–2.5cm cranial-caudal individualized margins. Boost volumes were definied with repeated CT scans if needed.

Plans were normalized to 95% of the planning target volume (PTV) (D_95%_=100%). For all patients, in addition to the target volume PTV, the entire liver, the normal liver (liver-PTV), the kidneys, the stomach, the spinal cord and the lungs were outlined and considered during optimization. The following explicit planning objectives were defined: for the total liver V_30Gy_<60%, for the normal liver (liver-PTV); V_15Gy_<30%, for the PTV a minimum dose greater than 90%; and a maximum dose lower than 115-140%.

All patients were treated with VMAT in the form of RapidArc [13,14] with 10 MV photons. Individualized optimization was performed using single or multiple, coplanar or non-coplanar, mono-isocentric arcs.

All patients were treated in supine position with arms placed overhead and were immobilized with an individualized vacuum cushion on the patient tray. A real-time infrared tracking device (ExacTrac; BrainLab AG, Heimstetten, Germany) was used for patient immobilization and reposition during the CT scan and treatment.

### Evaluation

Dosimetric and technical parameters of delivery were scored including some delivery parameters as well as standard analysis of dose volume histograms (DVH). Guidelines of International Commission on Radiation Units and Measurements (ICRU) 83 report were applied as far as possible [18]. Clinical evaluation was performed, with reference to baseline conditions determined before start of treatment, during treatment and at 1, 2, 3,6 months after treatment completion: basic treatment outcome was measured in terms of in-field local control (visits included laboratory assessment and CT and MRI imaging (at 2 to 3 month intervals for at least 2 years and at 6 month intervals thereafter)) and patient overall survival and it was scored continuously with a total follow-up of maximum 28 months. Tumor response was assessed using Response Evaluation Criteria in Solid Tumors (RECISTs) criteria. Local in field recurrence was defined by new enhancement or progressive disease with CT or MR imaging during follow-up. Actuarial survival and local control rates were determined by standard Kaplan-Meier analysis and several factors were tested to ascertain significant differences between subgroups of patients. Univariate and multivariate analysis were similarly performed to identify variables relevant for survival prediction. RILD (in absence of progressive liver disease), was defined by Lawrence’s criterion [7]. Conventional definition of classical RILD [9] manifested within 4 months after the completion of irradiation as either anicteric elevation of alkaline phosphatase level to at least two-fold of the upper normal level and nonmalignant ascites, is not common in Asia. Instead, non-classic RILD is dominant and GPT elevation indicates hepatocyte damage. Bujold [3] defined non-classical RILD as a 5-time elevation in liver transaminases. The significant liver dysfunction is often difficult to differentiate treatment-induced hepatic toxicity from progressive tumor or dysfunction exist prior to treatment such as cirrhosis or previous treatment. Thus, we defined RILD as elevated transaminase of at least two-fold the upper limit of normal or pretreatment levels based on the National Cancer Institute Common Toxicity Criteria for Adverse Events (CTCAE) version 4.03. Gastrointestinal (GI) toxicity was also scored according to CTCAE 4.03. Endpoints included esophagitis, gastritis, gastric hemorrhage or ulceration, duodenal hemorrhage or ulceration and ascites.

## Results

### Patients

Of the 138 patients treated with RapidArc included in the analysis, Twenty-nine patients were not assessed in terms of treatment response because either dead (21) or lost to follow-up (8). Table 1 reports the characteristics of the cohort of patients included in the study. Concerning stage, the vast majority of the cases presented advanced local diseases: BCLC stage C in 72.5% of the population, AJCC stage III and IV in 88.4% of the cases. About half of the cases were with portal vein thrombosis; 86.2% had history of hepatitis. For all patients, median tumor volume was 516 cm^3^ (ranging up to 3621cm^3^) corresponding to 33% of the median total liver volume.

**Table 1:** Characteristics of the cohort of patients

**Characteristics**	**Items**	**N (%)**
Sex	Female	26 (18.4%)
	Male	112 (79.4%)
Age	Mean	64
	Median (range)	66 (30–87)
	St.dev	11
Portal Vein Thrombosis	No	64 (46.4%)
	Yes	74 (53.6%)
Tumor location	Right lobe	57 (41.3%)
	Left lobe	10 (7.2%)
	Bilateral lobe	71 (51.4%)
Stage T	T1	8 (5.8%)
	T2	10 (7.2%)
	T3	120 (86.9%)
Stage N	N0	114 (82.60%)
	N1	24 (17.4%)
Stage M	M0	116 (84.1%)
	M1	22 (15.9%)
AJCC Stage	I	7 (5.1%)
	II	9 (6.5%)
	III	83 (60.1%)
	IV	39 (28.3%)
Okuda Stage	I	31 (22.4%)
	II	107 (77.6%)
BCLC Stage	A	9 (6.5%)
	B	29 (21.0%)
	C	100 (72.5%)
Child-Pugh Stage	A	96 (69.6%)
	B	42 (30.4%)
Hepatitis	No	19 (13.8%)
	B	71 (51.4%)
	C	43 (31.2%)
	B and C	5 (3.6%)
Initial Alpha-fetoprotein (?g/L)	Median (range)	11481 (2.4 – >58300)
Initial white blood count (kU/?L)	Median (range)	5.9 (2.7 – 15.6)
Initial haemoglobin level (g/dL)	Median (range)	12.8 (7.0 – 19.0)
Initial GPT level (U/L)	Median (range)	50.0 (8.8 – 396.0)
Initial total bilirubin level (mg/dL)	Median (range)	0.9 (0.3 – 8.5)
Initial tumor volume (cm^3^)	Median (range)	516 (28 – 3621)
Total liver volume (cm^3^)	Median (range)	1587 (548 – 5489)
Dose prescription	45Gy	16 (11.6%)
	60Gy	114 (82.6%)
	66Gy	8 (5.8%)

Values refer to number of patients, % to the total number of 138 patients.

AJCC: American joint Committee on Cancer, BCLC: Barcelona Clinic Liver Cancer. HCC: hepatocellular carcinoma. TACE: transarterial chemo-embolisation. RFA: radio-frequency ablation. GPT: Glutamic Pyruvic Transaminase.

### Treatment

Almost (88.4%) patients received either 60 or 66Gy and about 61% were treated with one or more volume reductions (cone-down). About 88% of the patients were treated with 2Gy/fraction, 12% with 1.8Gy/fraction. Nearly 99% of the patients were treated with multiple partial arcs and 93% were optimized with a non-coplanar setting.

Table 2 shows results from the dose-volume histogram (DVH) analysis for target volumes and organs at risk. The planning objective on V_30Gy_ for total liver and on V_15Gy_ for normal liver (liver-PTV) were on average respected together with a substantially full coverage of the PTV (V_95%_ and D_95%_ showed a minimum value of 96% and 96.8%).

**Table 2:** Summary of the DVH analysis for the target volume and for the organs at risk for the entire cohort of patients

**Organ**	**Parameter**	
PTV	D_95%_ [%]	100.8±6.2 [96.0;108.0]
	V_95%_ [%]	99.0±1.8 [96.8;100.0]
	Mean [%]	110.1±2.6 [101.7;120.6]
Left Kidney	Mean [Gy]	5.22±4.5 [0.2;21.2]
	D_1cm3_ [Gy]	12.3±8.3 [0.4;34.9]
Right Kidney	Mean [Gy]	8.2±7.2 [0.1;26.9]
	D_1cm3_ [Gy]	28.1±18.7 [0.3;62.6]
Spinal Cord	D_1cm3_ [Gy]	21.8±9.0 [1.1;47.6]
Stomach	Mean [Gy]	14.6±5.7 [3.0;36.6]
	D_1cm3_ [Gy]	31.7±10.9 [8.5;58.2]
Liver-PTV (Normal liver)	Mean [Gy]	19.4±6.3 [1.9;33.4]
	V_15Gy_ [%]	24.4±11.6 [0.6;56.4]
Total Liver	Mean [Gy]	32.1±11.5 [2.3;55.4]
	V_30Gy_ [%]	46.4±19.7 [1.4;85.4]

Data are reported as average values plus or minus standard deviation; in square brakets the range.

Dx%: dose received by at least x% of the volume.

Vx%: volume receiving at least x% of the dose.

### Survival

**Figure 2:**
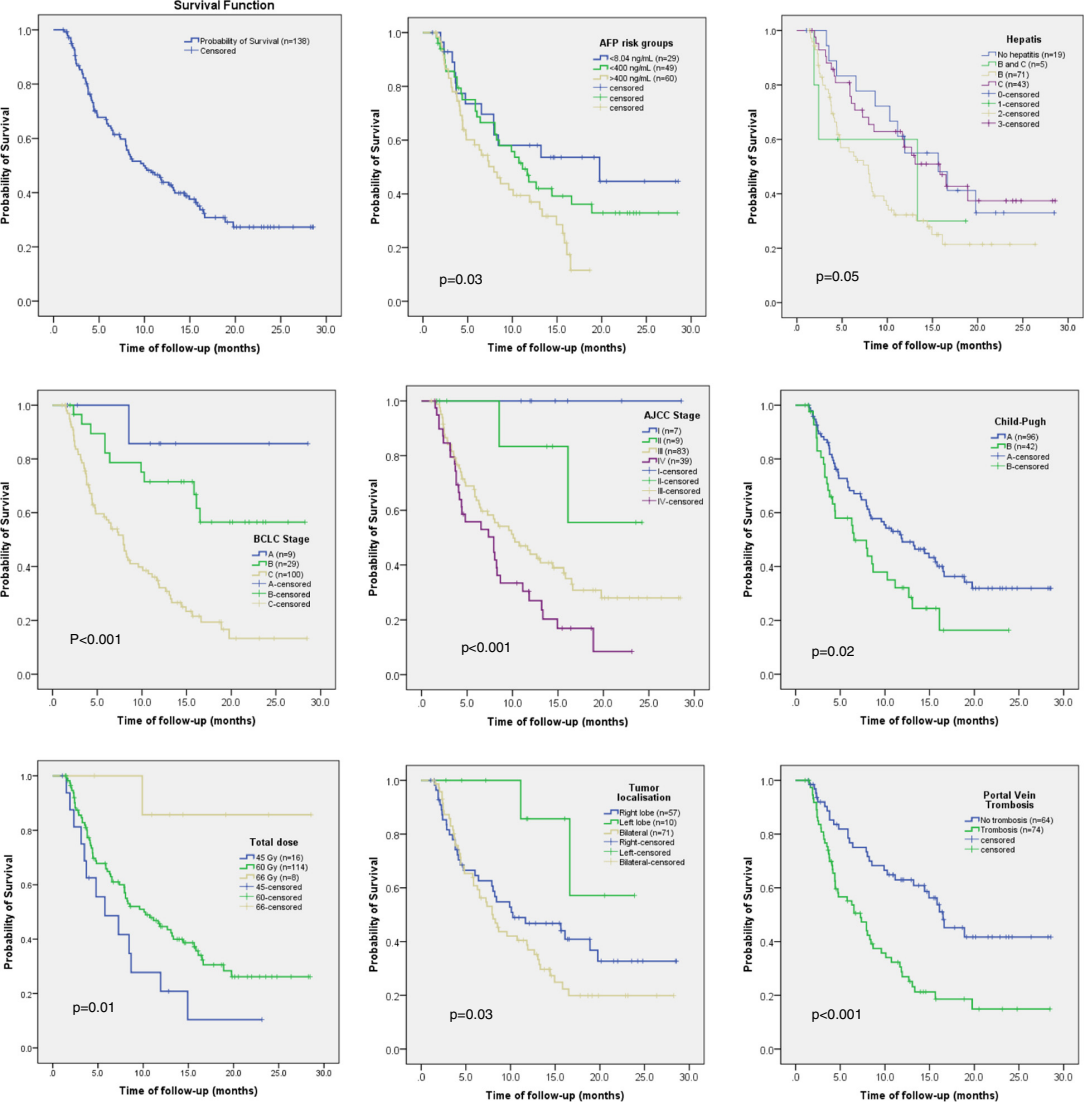
**Survival plots for the whole cohort of patients and factorized according to some significant variables** .

Figure 2 shows survival estimates after Kaplan-Meier analysis for the whole cohort of patients (n=138) and factorized according to some of the variables leading to significant differences. Mean survival time resulted 13.5 months (95% C.I.: 11.6–15.3 months), median resulted 10.3 months (95% C.I.: 7.2-13.3 months). Overall survival (OS) at 12 months resulted 45±5%, at 24 months 28±5%. One-year OS based on AJCC stage was: stage I: 100%; stage II: 83%; stage III: 45%; stage IV: 28%. The same OS classified by BCLC is: stage A 85.1% (4 patients alive), B 74.1% (19 patients alive), C 32.5% (24 patients alive). Table 3 summarizes the mean OS time factorized according to some of the main significant variables. Univariate analysis lead to the result that the following variables are significant with respect to survival (in parenthesis the significance): sex (p=0.05), age (p=0.006), total dose (p=0.01), tumor volume (p<0.001), localization (p=0.03), portal vein thrombosis (p<0.001), AJCC stage (p<0.001), BCLC stage (p<0.001), normal liver volume (p<0.001), T stage (p<0.001), baseline alpha fetoprotein (AFP) level at before therapy (p=0.03), volume of total liver receiving more than 30Gy (p=0.001). In a multivariate analysis, the following variables resulted statistically significant: total liver volume (p=0.001), tumor volume (p=0.002), total liver volume irradiated more than 30Gy (p=0.02), portal vein thrombosis (p=0.001), T stage (p=0.001), AJCC stage (p=001), BCLC stage (p<0.001) and baseline alpha fetoprotein level (p=0.02). Survival did not resulted significantly different (p=0.11) if factorized according to Okuda stage I and II.

**Table 3:** Summary of the mean probability of survival factorized according to significant variables

**Factor**	**Probability of survival (months) [95% C.I.]**	**P value**
Sex	Male: 13.1 [11.1 – 15.1]	0.05
	Female: 15.2 [11.4 – 19.0]	
Portal vein thrombosis	No: 17.7 [14.9 – 20.5]	<0.001
	Yes: 10.1 [7.9 - 12.3]	
BCLC stage	A: 25.7 [20. – 30.9]	<0.001
	B: 20.1 [16.3 – 23.9]	
	C: 13.5 [11.7 – 15.4]	
Child-Pugh	A: 14.8 [12.6 – 17.1]	0.02
	B: 9.9 [7.3 – 12.6]	
Hepatitis	No: 16.6 [12.1 – 21.1]	0.05
	B: 10.9 [8.6 – 13.3]	
	C: 16.3 [12.9 – 19.7]	
	B&C: 10.5 [4.1 – 16.8]	
Baseline AFP	Baseline: 17.2 [12.8 – 21.6]	0.03
	Low risk:14.6 [11.5 – 17.7]	
	High risk:9.3 [7.7 – 10.9]	
Tumour localisation	Right lobe: 14.6 [11.5 – 17.6]	0.03
	Left lobe: 20.0 [15.7 – 24.3]	
	Bilateral: 11.8 [9.4 – 14.2]	
Total dose	45 Gy: 8.6 [5.1 – 12.1]	0.01
	60 Gy: 13.5 [11.4 – 15.5]	
	66 Gy: 25.9 [21.1 – 30.7]	
Volume of normal liver Receiving >15Gy	<30%: 21.0 [17.6 – 24.4]	0.001
	>30%: 18.6 [16.9 – 20.4]	

In brakets the 95% confidence interval level.

### Response and toxicity

The mean time to local response was 8.8 months (95% C.I.: 7.9–9.7 months). The responses were: progressive disease (PD): 6.4% (7 of 109 assessed patients) with a mean time to progression detection of 3.1±2.3 months (range 1.1 to 7.2 months); stable disease (SD): 29.4% (32 patients) with mean time to detection of 4.0±1.3 months (range: 1.4-6.8 months); partial response (PR): 53.2% (58 patients) mean time to response of 4.6±1.3 months (range: 1.2-8.9 months); complete response (CR): 11.0% (12 patients), mean time to complete response of 4.9±1.7 months (range: 3.0-9.8 months). Twenty-nine patients were not assessable as described above. Hundred and two patients responded with progression-free (SD+PR+CR), 93.5% of the assessed group or 73.9% of the entire cohort of 138 patients.

AFP level was monitored and its reduction from baseline (before treatment) to the end of radiotherapy resulted highly significant for the entire cohort of patients (20% reduction, p<0.01). Total reduction and significance remained high at 1, 3 and 6 months (reduction of 27%, 27% and 29%; p=0.01, p<0.01 and p=0.03). Different trends were observed for the two risk groups (baseline AFP between normal level to 400ng/L versus greater than 400ng/L). For the first risk group, AFP increased during treatment and follow-up: +22% at end of treatment, +173% at 1 month after end of treatment and +228% at 3 months with high significance of differences. The patients belonging to the highest risk group, showed, on the contrary, a remarkable reduction of AFP: 20% at the end of treatment, 29% at one and 3 months (p<0.01, p<0.01, p=0.03). The relative majority of PR or CR (29/70) were observed in the AFP highest risk group. No impact was observed on total bilirubin levels between baseline and end of therapy. A significant drop in WBC was observed between baseline and end of therapy (p=0.01), partially recovered but still significant at 3 and 6 months. For hemoglobin levels, the trend is similar with WBC.

Concerning treatment toxicity, non-conventional RILD was observed in 34 cases (25% of 138 patients), but the most cases (24/34) were grade 1 or 2 toxicities (Table 4). Four patients with severe RILD (grade 3 or 4) were observed in the AFP first risk group (AFP from 8.04 to 400ng/mL) and 3 in each of the normal level or high risk groups. GI toxicity is summarized in Table 4 and resulted mild. For each endpoint grade 3 was observed in 1-2% of cases while grade 1–2 ranged from 2% (esophagitis) to 23% (ascites).

**Table 4:** Summary of toxicity profiles based on CTCAE criteria

	**Gr 1**	**Gr 2**	**Gr 3**	**Gr 4**
RILD	12 (9%)	12 (9%)	8 (6%)	2 (1%)
Esophagitis	1 (1%)	2 (1%)	2 (1%)	-
Gastric Hemorrhage	0 (0%)	8 (6%)	3 (2%)	-
Gastric Ulcer	1 (1%)	17 (12%)	3 (2%)	-
Gastritis	4 (3%)	11 (8%)	1 (1%)	-
Duodenum Hemorrhage	0 (0%)	4 (3%)	3 (2%)	-
Duodenum Ulcer	3 (2%)	13 (9%)	3 (2%)	-
Ascites	11 (8%)	21 (15%)	2 (1%)	-

## Discussion

The current study demonstrated the possibility to treat HCC patients with RapidArc safely and effectively within a conventional fractionation scheme and total dose ranging from 45 to 66Gy. Patients treated were mostly in advanced stages (>90% in BCLC stages B and C) with frequent presence of portal vein thrombosis. Early results in terms of overall survival and local control demonstrated a high level of local control (94% of the assessable patients) and the overall survival at 12 months resulted 45% (significantly higher for earlier stages). A significant clinical effect at biochemical level was observed in the decline of the AFP levels at the end of treatment and during follow-up with a 30% reduction of AFP for the whole cohort and of more than 50% for the patients with poor baseline.

The application of non-coplanar rotational technique with intensity modulation is a unique characteristic in this study. RapidArc allowed to spare normal liver tissue and led to be an acceptable treatment option for those patients ineligible for surgery or other ablative treatments. Because of the advanced stage, it was not possible to add also hypo-fractionated regimen for these HCC patients. Therefore, although this modality has better or similar effect in tumor response as the standard treatment, any comparison against other published studies has to account for the trade-off between the innovative technique and the limits of conventional fractionation. Given the short follow-up, results might be time-biased (local control might be overestimated or toxicity under-estimated) in present study. However, it is important to position the new treatment technique in the frame of consolidated experience of other groups. Hsu [19] reported about bi-fractionated treatments with conformal therapy: from a pool of 121 patients, 1 year survival was 60%. Krishnan [20] reviewed studies of radiotherapy in liver including typically TACE, 1 year survival ranged from 42% to 94% for doses ranging from 30 to 66Gy. Seong [21] demonstrated 158 patients treated with conventional fractionation scheme and in combination with TACE. One-year and 2-year OS was 40% and 20% with a median survival of 10 months, respectively. Seong also found that total dose was the only significant factor influencing survival and the mean prescribed dose was 48Gy. Data from this study demonstrated a similar effect with a huge difference between the three-dose levels administered (although masked by several other prognostic factors in the dose level assignment). Seong reported comparable results in terms of local control compared to this study. Skinner [22] reported a small cohort of 29 patients demonstrating once more the relevance of total dose to improve outcome. Patients treated with lower total doses (biological effective dose <75Gy) resulted in 1-year OS of only 18% against 69% for the complementary group. Given these perspective and the results of the present study, it is conceivable to escalate the dose prescribed with RapidArc also for advanced stage patients with HCC. Yoon [23] analyzed clinical outcome for 412 patients treated with TACE and 3D conformal radiotherapy for HCC with portal vein thrombosis. For these patients, median survival was 10.6 months with 42.5% survival rate at one year consistent to the data reported in the present study (35% survival for PVT positive patients).

This study also allowed to identify some correlation between outcome and AFP levels before treatment. Patients within the highest risk group of AFP had the most significant reduction of the same during follow-up. Although no definitive answer can be provided here, one possible interpretation, might be related to the fact that non-significant AFP elevation will occur when either hepatitis reactivation or change of cirrhosis status will occur after radiotherapy. Thus, for those patients with lower AFP levels (first risk group), the AFP might be increasing because of the effects of inflammation/cirrhosis are higher than therapeutic effect in the early follow-up period. On the contrary, for those patients with higher AFP levels (high risk group), even assuming a similar proportion of tumor cell killing as within the first group, the absolute AFP values will decrease more.

Finally, an advantage of RapidArc is its effective ‘rapid’ delivery time. This might overcome most of the problem of respiratory motion in abdominal tumours, especially in HCC patients. In particular, conventional respiratory gating may not be feasible for HCC patients (breath hold or deep inspiration are impractical for the patients with huge HCC and massive ascites). While for most advanced stages, respiratory motion could be automatically accounted for by the extension of margins, for earlier stages, the usage of time resolved CT scan (4DCT) for planning might help to understand the liver excursion and to better individualise the margins to PTV. This is particularly important in those cases where dose escalation might be envisaged.

## Conclusion

The cohort of 138 patients suffering of HCC was treated with VMAT. Early results in terms of OS and local control were adequate and consistent with similar reports demonstrating the appropriateness of the RapidArc technique for advanced HCC patients. Toxicity profile was mild. Dose escalation might be considered to improve survival, either increasing the total dose or, for earlier stages, considering the SBRT solution. Longer follow-up will allow to consolidate these early observations.

## Competing interests

Dr. L. Cozzi is Head of Research at Oncology Institute of Southern Switzerland and acts as Scientific Advisor to Varian Medical Systems.

## Authors' contribution

PMW and LC coordinated the entire study. Data collection was conducted by PMW, WCH, NNC, FLC. Data were analyzed by PMW, WCH, NNC, FLC, AF, LC. All authors read and approved the final manuscript.
